# USMCA (NAFTA 2.0): tightening the constraints on the right to regulate for public health

**DOI:** 10.1186/s12992-019-0476-8

**Published:** 2019-05-14

**Authors:** Ronald Labonté, Eric Crosbie, Deborah Gleeson, Courtney McNamara

**Affiliations:** 1 School of Epidemiology and Public Health, University of Ottawa, 600 Peter Morand Crescent, Ottawa, Ontario K1G 5Z3 Canada; 2 School of Community Health Sciences, Ozmen Institute for Global Studies, University of Nevada Reno, 1664 N. Virginia Street, Reno, NV 89557-0274 USA; 3 School of Psychology and Public Health, La Trobe University, Bundoora, VIC 3086 Australia; 4 Department of Sociology and Political Science, Norwegian University of Science and Technology, NO-7491 Trondheim, Norway

**Keywords:** Trade and investment policy, Public health, NAFTA, USMCA, Regulatory coherence, TRIPS-plus, Labour and environmental protection

## Abstract

**Background:**

In late 2018 the United States, Canada, and Mexico signed a new trade agreement (most commonly referred to by its US-centric acronym, the United States-Mexico-Canada Agreement, or USMCA) to replace the 1994 North American Free Trade Agreement (NAFTA). The new agreement is the first major trade treaty negotiated under the shadow of the Trump Administration’s unilateral imposition of tariffs to pressure other countries to accept provisions more favourable to protectionist US economic interests. Although not yet ratified, the agreement is widely seen as indicative of how the US will engage in future international trade negotiations.

**Methods:**

Drawing from methods used in earlier health impact assessments of the Trans-Pacific Partnership agreement, we undertook a detailed analysis of USMCA chapters that have direct or indirect implications for health. We began with an initial reading of the entire agreement, followed by multiple line-by-line readings of key chapters. Secondary sources and inter-rater (comparative) analyses by the four authors were used to ensure rigour in our assessments.

**Results:**

The USMCA expands intellectual property rights and regulatory constraints that will lead to increased drug costs, particularly in Canada and Mexico. It opens up markets in both Canada and Mexico for US food exports without reducing the subsidies the US provides to its own producers, and introduces a number of new regulatory reforms that weaken public health oversight of food safety. It reduces regulatory policy space through new provisions on ‘technical barriers to trade’ and requirements for greater regulatory coherence and harmonization across the three countries. It puts some limitations on contentious investor-state dispute provisions between the US and Mexico, provisions often used to challenge or chill health and environmental measures, and eliminates them completely in disputes between the US and Canada; but it allows for new ‘legacy claims’ for 3 years after the agreement enters into force. Its labour and environmental chapters contain a few improvements but overall do little to ensure either workers’ rights or environmental protection.

**Conclusion:**

Rather than enhancing public health protection the USMCA places new, extended, and enforceable obligations on public regulators that increase the power (voice) of corporate (investor) interests during the development of new regulations. It is not a health-enhancing template for future trade agreements that governments should emulate.

## Background

On November 30th 2018, the United States, Canada, and Mexico signed their ‘NAFTA 2.0’ agreement, generally referred to as the USMCA or, in Canada, the CUSMA and, in Mexico, the T-MEC (each country is entitled to position itself first when referencing the agreement). Negotiation of the agreement was strained and took place in the context of the US Trump Administration’s imposition of steel and aluminum tariffs affecting both Canadian and Mexican manufacturing. In doing so, the US invoked a ‘national security’ exemption under World Trade Organization (WTO) rules, subsequently challenged under WTO dispute settlement rules, but likely years away from resolution. Trade war pressures emanating from the Trump Administration are widely seen as leading to a ‘NAFTA 2.0’ that is largely beneficial to US corporate and political interests, although both Canada and Mexico were able to protect some of their own interests.

The three countries have deeply integrated economies: Canada and Mexico are the top two trading partners of the US, while Canada and Mexico rank third with each other. The total value of trade in goods and services between the three countries in 2017 was USD 1.3 trillion, USD 673 billion in US/Canada trade, USD 616 billion in US/Mexico trade [1], and USD 33 billon in Canada/Mexico trade [2]. Economic reliance on exports to the US, however, is greater for both Canada and Mexico than the reverse, given the much larger size of the American domestic economy. All three countries have made positive claims about the new agreement, emphasizing its economic benefits to their own economies while also claiming improved labour and environmental protection measures.

To the extent that economic growth consequent to the agreement is both equitable and ecologically sustainable, it will have knock-on positive health impacts. Whether this is likely to be the case is a matter of ongoing debate. There are also palpable concerns that the agreement’s new measures on intellectual property and regulatory coherence will limit the public health policy space of all three countries or, at best, will lead to regulations in Canada and Mexico more closely aligned with those in the US which, at the national level, is currently favouring corporate self-regulation over government legislation. At this point in time (May 2019) the USMCA has yet to be ratified by all three Parties. Although this is expected to occur sometime later in the year, it is still possible that push-back on the agreement in the US, from both political parties, could prevent it from entering into force in its current form.

In this article we assess the health implications of the USMCA following the methods and approach we used in our earlier health impact assessment (HIA) of the Trans-Pacific Partnership Agreement (TPP) [3–6] (now the CPTPP following the US withdrawal from the agreement in early 2017). We begin by discussing how the USMCA’s provisions on intellectual property rights (IPRs) are likely to affect access to medicines, before turning to multiple new measures in the agreement that are likely to diminish government’s autonomous policy space. Changes to investment protection rules (often used to challenge environmental or other public health measures) herald a more positive health outcome, but will continue to allow for foreign investor suits against new government regulations considered to be an ‘expropriation’ of the value of their investment for several more years. We then turn to the claimed improvements in labour and environmental protection. Throughout we use the WTO and the CPTPP agreements as comparators to highlight changes in the USMCA. We conclude with a discussion of what the USMCA portends for the future of new regional or bilateral free trade agreements (FTAs), and the future of the multilateral trading system (the WTO).

### Pharmaceuticals and access to medicines

Several chapters and annexes of the USMCA have implications for pharmaceutical policy and access to medicines. Consistent with the well-documented US ‘template approach’ to negotiating trade agreements, where each trade agreement negotiated builds on the last [7], the USMCA text relevant to pharmaceuticals generally takes the TPP text as its starting point [8]. In some areas the commitments are broadened and/or deepened in comparison with the TPP, and the USMCA also reinstates some TPP provisions that were suspended in the CPTPP following the withdrawal of the US.

The most significant chapter in terms of access to medicines is Chapter 20 (Intellectual Property Rights). This chapter includes many of the ‘TRIPS-Plus’ rules^1^ from the TPP as originally negotiated, including the following obligations, which were subsequently suspended in the CPTPP:

- the requirement to make patents available for ‘new uses of a known product, new methods of using a known product, or new processes of using a known product’ (USMCA Art. 20.36 para 2), which facilitates ‘evergreening’ of pharmaceutical patents;
- patent term adjustment (i.e. extension) for ‘unreasonable’ granting authority delays (Art. 20.44) and for ‘unreasonable or unnecessary’ delays in the marketing approval process (Art 20.46) – mechanisms for lengthening the patent term beyond 20 years for many drugs;
- protection of undisclosed test or other data submitted to regulatory authorities (Art. 20.48), preventing regulators from using the clinical trial data submitted by the originator company to assess an application from a generic company for a period of time (at least 5 years for new pharmaceutical products, and either an additional 3 years for test data submitted to support a new use or formulation, or 5 years for combination products including a drug that has not previously been approved);
- a 10 year period of ‘effective market protection’^2^ for biologics (medicines produced from living cells and other biological materials via biotechnology processes, which include many new cancer and immunotherapy drugs) (Art. 20.49), the longest period of market protection for such drugs negotiated in a trade agreement to date [8, 9].

In addition to reinstating these suspended TPP provisions, the USMCA (Art. 20.51) also reproduces the CPTPP’s ‘patent linkage’ obligation, which establishes a link between the patent status of medicines and the marketing approval process, potentially delaying the market entry of generics while disputes over possible patent infringement are resolved.

There is a large body of research tracing the implications of these types of TRIPS-Plus provisions for access to medicines, which all serve to delay the market entry of less expensive generic and biosimilar^3^ medicines (see, for example [10–12]). Similar to the TPP (and other recent regional or bilateral trade agreements) the USMCA recognizes the flexibilities afforded in the 2001 Declaration on TRIPS and Public Health (Art.20.A.6) which allows for compulsory licensing of generic versions of patented drugs, thereby lowering their costs. Use of compulsory licensing remains limited, however, particularly for medicines other than antiretroviral drugs [13, 14], and the use of this TRIPS flexibility is likely to continue to face fierce opposition from countries with sizeable research-based pharmaceutical industries, especially if invoked for biologics.

Indeed, the extended protection for biologics, which are expected to comprise almost 28% of the global pharmaceutical market by 2020 [15], is of particular health concern. Biologics are currently eligible for 8 years of market protection in Canada (with an extension of 6 months for drugs where tests have been undertaken in children), so the change required to implement the USMCA will be incremental. In Mexico, however, the effect will be more dramatic, as biologics are not currently subject to data protection [8]. In this context, any delays to the market entry of biosimilars would come at a high cost. The actual impact of extending market protection is difficult to ascertain, as the market protection period commences on the date of marketing approval; in many cases the patent protection will extend beyond the market protection period. A recent Canadian Parliamentary Budget Office review estimated that the USMCA patent market protection provisions for biologics would increase drug costs by as much as CDN$169 million by 2029, increasing each year thereafter [16]. Another study predicted annual lost savings in the range of $0 to $305.8 million depending on whether patents would expire or be challenged prior to the end of the market protection period [9].Although the number of drugs affected may be small, the cost impact for each may be large: in Canada, for example, potential savings from biosimilars are estimated at between 8 and 43% of the cost of the original drug [7]. In the US, the USMCA biologics provisions would stymie attempts to wind back the current 12 year market exclusivity period for biologics to 7 years, as proposed in legislation introduced to Congress in 2017 to improve access to medicines [17].

Beyond the intellectual property chapter, a number of other chapters and annexes of the USMCA have implications for access to medicines. Possibly the most significant of these is Chapter 29 (Publication and Administration) which includes a set of rules applying to national programs for listing pharmaceuticals for reimbursement. These provisions are almost identical to Annex 26-A of the TPP. The detailed procedural rules which these programs must follow were suspended in the CPTPP after the US withdrew, but have been incorporated once again into the USMCA. Neither Canada nor Mexico currently have a national pharmaceutical program which is covered by the rules (which are also not enforceable through state-state dispute settlement). If either country introduces one in the future, however, it will be expected to comply with a set of rules that may limit flexibility in decision making about which drugs to subsidise and when, and will require avenues for pharmaceutical company input to the process [9].

Finally, there are a number of other parts of the USMCA text which touch on other aspects of pharmaceutical policy aside from access to medicines. An example is Annex 12-F (Pharmaceuticals), which sets out detailed procedural requirements for marketing approval processes and pharmaceutical inspections. This is very similar to Annex 8-C of the TPP, which includes provisions that may lower standards for assessing drug safety and efficacy, increase pressure to speed up regulatory processes (with concomitant safety risks), and place constraints on the public release of information about pharmaceutical inspections [9].

### Regulatory policy space

Policy space refers to how international treaties or global trade rules can affect the flexibilities of national, state/provincial, and local governments to enact domestic policies, regulations, or other measures. The potential for trade agreements to impinge upon governments’ abilities to regulate in the interest of health, environmental, and social protection has long been a concern amongst trade policy analysts [3–6, 18]. The preamble to the USMCA attempts to assuage concerns about the agreement’s impact on regulatory policy space by affirming the “inherent right to regulate and resolve to preserve the flexibility of the Parties to set legislative and regulatory priorities*, in a manner consistent with this Agreement*”. The italicized phrase, consistent with other recent agreements such as the TPP, essentially requires government measures to abide wholly with the rules set out in the USMCA. This perforce requires a detailed look at what those new rules mean. Although the preamble continues to state that Parties are able to “protect legitimate public welfare objectives, such as public health, safety, the environment, the conservation of living or non-living exhaustible natural resources, the integrity and stability of the financial system and public morals,” once more this must be “in accordance with the rights and obligations provided in this Agreement”. We return to this issue later in our paper when we discuss permissible exceptions to USMCA trade rules.

#### Agriculture

The first regulatory concern appears in Chapter 3 (Agriculture), an issue of importance to all three Parties. Canada and Mexico rank first and third as markets for US food and agricultural exports [19], while almost 80% of all Mexican agriculture exports go to the US [20]. The legacy of NAFTA 1.0, however, was devastating for Mexican farmers. Under NAFTA rules Mexico was required to eliminate tariffs over a 15 year period on heavily subsidized agricultural imports from the US. Corn tariffs were eliminated much more rapidly, resulting in a sudden, sharp drop in corn prices in Mexico and ‘dumping’ of heavily subsidized US corn products [21]. NAFTA did not eliminate such subsidies, with US subsidies on agricultural exports to Mexico rising dramatically while Mexico’s (considerably weaker) agricultural subsidies going primarily to large commercial producers rather than to small-scale farmers [22]. These trade-related policy shifts lead to nearly 5 million rural farmers being displaced between 1991 and 2007, with 3 million of these moving from independent farming to (primarily) seasonal farm labour. Unsurprisingly, Mexican rural poverty rates were much the same in 2010 as when NAFTA came into effect; while agricultural market liberalization, to which NAFTA 1.0 contributed, is considered by some to be the source of the first wave of mass migration of Mexicans to the US, in search of employment [23], a migration partly but not fully obviated by government social programs and income transfers intended to dent rural poverty [22].

US exports of corn to Mexico in 2017 still outstripped Mexican exports of corn to the US by a factor of 11:1 [20]. What is striking, however, is the progressive shift towards industrial-scale farming in all three countries which, while not caused by trade agreements per se, are enabled and accelerated by such market liberalization rules. In Canada and the US, the percent of population reliant on agriculture fell from 4.26 in 1994 to just 1.9 in 2018 (Canada), and from 2.8 to 1.6 over the same period (US). Mexico also suffered a decline, from 27% in 1994 to just 13% in 2018 – but still a considerably higher dependency ratio than its two agricultural trading partners. Any agricultural employment loss consequent to USMCA rules will be experienced most harshly by rural Mexicans, with probable knock-on negative health impacts.

There are two specific and long-standing concerns with agriculture affected by new measures in the USMCA. The first applies to the role of domestic subsidies to producers which, under terms of the WTO’s Agreement on Agriculture, have allowed high-income countries such as the US, Japan, and the European Union (EU) to continue underwriting their own farming industry to the detriment of competing producers in low-income countries unable to afford similar levels of support. Attempts to fully resolve this at the WTO have so far failed. The USMCA commits Parties to work together at the WTO, “with the objective of substantial progressive reductions in agriculture support and protection” (Art.3.3.), which may be little more than rhetorical flourish but which also ignores that many low- and middle-income countries want to retain certain levels of domestic support for their own farmers for purposes of poverty reduction, essential to improving equitable health outcomes. Instead, the agreement requires that any support to its agricultural producers “shall consider domestic support measures that have minimal or no trade distorting or production effects” (Art.3.6.1). “Shall consider” is not binding language and relies upon a consultation process in cases where trade distortion is declared. Whether or not this provision, if ratified, will have any impact on US support to its agricultural exports, where for the past 3 years alone its corn and wheat exports to Mexico have been at 10 and 32% below production costs, respectively [24], remains an open question. It could also negatively affect recent (post USMCA signing) commitments by the new Mexican government to achieve greater self-sufficiency in food production and reduce rural poverty by guaranteeing minimum prices for a number of basic food commodities [25].

Food security is a second, related concern. Art. 3.9 prevents Parties from utilizing existing WTO special agricultural safeguards, which would allow them to enact temporary trade barriers in cases of import surges or unstable prices which could affect their own future capacities to produce food, thereby increasing their reliance on exports from other Parties. Once more, this is likely to affect farmers in Mexico to a much greater extent than those in the other two countries with substantially lower rates of farm labour reliance. Art.3.5 places further requirements for “transparency and consultation” over the use of WTO safeguard measures to restrict food exports when faced with a “critical shortage of foodstuffs”. It is hard to pre-determine how this WTO+ provision might play out between the three countries, although it was considered a sufficiently important “win” to be singled out as such by the US Trade Representative Office [26]. An additional potential burden on food security requires Parties to ratify the 1991 version of the International Union for the Protection of New Varieties of Plants (UPOV 1991), which prohibits farmers from saving and sharing seeds of protected varieties (Art.20.A.7 [2]). Mexico ratified an earlier version of UPOV in 1978, which allows exceptions for small-scale farmers, but declined to ratify the strict UPOV 1991 version. Since the same provision already exists in the CPTPP, to which both Mexico and Canada are Parties, this provision will not change practices in either country, but constitutes a further strengthening of IPRs on genetically altered agricultural products.

The Annexes to Chapter 3 impose further regulatory restrictions. The US/Mexico Annex, for example, requires Mexico to give equal treatment to “sugar or syrupy products” coming from the US, which is predominantly high-fructose corn syrup (HFCS). Some research finds that HFCS poses greater health risks than other forms of sweeteners, and that its increased use (driven primarily by US exports) is associated with higher rates of obesity, cardiovascular disease, and diabetes [27–29]. A recent study of NAFTA 1.0 found that tariff reductions on food and beverage syrups containing HFCS were associated with a 41% increase in kilocalorie per capita increase in sweetener supply in Canada; other matched OECD countries that did not have trade agreements with the US did not experience any such rise [30]. Annex 3-C includes provisions allowing suppliers of wine and distilled spirits to display information required by an importing country (including health information) on a supplementary label, such as a sticker applied to the container after importation. Like similar provisions in CPTPP Annex 8-A, these rules could frustrate efforts to introduce prominent, effective health warning labels on alcohol containers if they are interpreted as meaning that required information should not interfere with the main labels [30]. The USMCA text appears to further restrict policy space by specifying that supplementary labels should “not conflict with information on an existing label” (Annex 3.C.3.6), more strongly reinforcing the notion that mandatory information should be relegated to an unused space on the container.

#### Technical barriers to trade (TBT)

As with the CPTPP, the USMCA imports the basic rules of the WTO TBT agreement, but adds more requirements that reduce the regulatory flexibilities of the three countries or, more accurately, brings them into closer alignment with regulatory policies of the US. Both the WTO TBT agreement and the CPTPP defer to *Codex*, a WHO/FAO administered body, in the setting of international standards, requiring scientific risk assessments and a ‘necessity test’ if regulations exceed those agreed upon at *Codex* [4]. The CPTPP reduces some of the policy space for regulations exceeding international standards permitted under WTO rules [4], while the USMCA adds further restrictions. Art.11.4, for example, allows for wider, enforceable language on the recognition of national public or private standardization bodies as relevant international standards, which could extend to accepting voluntary standards (e.g. corporate standards that have been developed in the US) as equivalent to *Codex* standards for the purpose of developing national regulations. Like the CPTPP, the USMCA requires Parties to “cooperate with each other… to ensure that international standards, guides, and recommendations that are likely to become a basis for technical regulations…do not create unnecessary obstacles to international trade” (Art.11.4.4). This means that new standards should a priori be least trade restrictive, which risks subordinating issues of health and safety to that of trade.

This risk is more likely given the USMCA’s deference to the WTO’s TBT Committee Decision on International Standards (Art.11.4.5), which defines six principles for setting international standards. The problematic principle from a health or safety vantage is principle 4 (effectiveness and relevance) which states:

In order to serve the interests of the WTO membership in facilitating international trade and preventing unnecessary trade barriers, international standards need to be relevant and to effectively respond to regulatory and market needs, as well as scientific and technological developments in various countries. They should not distort the global market, have adverse effects on fair competition, or stifle innovation and technological development [31].

This is unenforceable under WTO rules (it is a Committee recommendation and not a binding agreement), but has become enforceable under the USMCA. As with other provisions on international standards, it sets trade, if not above, then at least competing with health and safety regulations. The USMCA further requires Parties to consider all possible international standards in devising their own and, if not considering a particular standard, they must provide a reason for not doing so (Art.11.4.5.3). This could see a government accept an international standard with a lower threshold of safety rather than one providing greater protection.

The agreement also requires mandatory use of regulatory impact assessments for any major new regulation it proposes to adopt (Art.11.5) with a provision allowing “persons” (which includes private corporate actors) from another Party to directly petition a Party’s regulatory authorities if it believes a less trade-restrictive method exists (Art.11.5.4). There is also a unique requirement whereby Parties must ensure that “technical regulations concerning labels… do not create unnecessary obstacles to trade” (Art.11.5.8). This could influence front-of-pack nutrition labeling, discussions on standards for which are presently underway at *Codex*. Already the US-based North American Meat Institute and Canadian Meat Council have complained that Canada’s front-of-pack interpretive labeling (similar to that in Chile and several other countries) violates the USMCA’s rules on good regulatory practice [32]. Moreover, if the *Codex* guidance emphasizes a single regional labeling regulation (at present it recommends a national or regional approach, but that coexisting labeling schemes should not contradict each other), there will be considerable pressure on Canada and Mexico to adopt standards preferred by the US food industry and likely less influential in shaping healthier food choices [33, 34].^4^

Art.11.7.22, in turn, imposes a long list of obligations on Parties introducing a new regulations with the potential to have “a significant impact on trade,” drawing on text from the CPTPP but adding additional requirements that could delay introduction of a new health or safety regulation, especially if there is only limited evidence available on its impact. Unlike the WTO TBT agreement, the USMCA requires a Party developing a standard or regulation to “allow persons of another Party to participate in no less favorable terms than its own persons in groups or committees of the body that is developing the standard” (Art.11.7.8), a provision that also goes further than the CPTPP in specifying that they must be given an equal opportunity to occupy a seat at the table. As we cautioned in our HIA of the TPP/CPTPP, these types of obligations increase the risk of regulatory capture by vested private interests.

The chapter on TBT also contains several sectoral annexes which further reduce government’s regulatory space. Regarding cosmetics, for example, which can pose health risks (e.g. [35, 36]), no importing Party “shall require…a marketing authorization for a cosmetic product, unless a Party identifies a human health or safety concern, *and a no less trade restrictive alternative is reasonably available, such as notifications and post-market surveillance, to effectively address the risks at issue*” (Art.12.B.5.3). The italicized text essentially obliges Parties to wait for problems to show up and try to remedy after-the-fact, a much more restrictive measure than would apply under other TBT agreements or the USMCA chapter itself. At the same time, it also prohibits “notification numbers” on cosmetic product labels, limiting post-market surveillance efforts.

#### Sanitary and Phytosanitary measures (SPS)

The opening Articles in this chapter are almost the same as the CPTPP which, in turn, are almost the same as in the WTO SPS agreement. The USMCA, however, adds two new novel requirements requiring Parties to “enhance compatibility of sanitary or phytosanitary measures as appropriate; and advance science-based decision making” (Art.9.3). While not per se a risk to SPS regulation, these do signal potential for ‘chill’ in the absence of whatever might be construed as science-based to support a novel regulation. The chapter restates the preamble that “Each Party has the right to adopt or maintain sanitary and phytosanitary measures necessary for the protection of human, animal or plant life or health,” but then immediately weakens this by adding the caveat, “provided that those measures are not inconsistent with the provisions of this Chapter” (Art.9.6). Any standard exceeding that recognized as an international standard (which de facto would bring it into accord with the SPS Chapter and would not be subject to dispute) would need to undergo “an assessment, as appropriate to the circumstances, of the risk to human, animal or plant life or health” a more loosely worded requirement than that found in the CPTPP:

The USMCA, like the CPTPP, does allow for provisional regulation in the absence of sufficient “relevant scientific evidence,” but adds a new requirement: that within a reasonable time a Party must seek “additional information necessary for a more objective assessment” (Art.9.6.5). Thus the USMCA (compared to the CPTPP) loosens the scientific noose in one SPS article, but then tightens it with another. Moreover, in the absence of measures that exceed an international standard, Parties will need to “provide an explanation of the reasons and pertinent relevant information regarding the measure upon request” if another Party believes the measure may constrain its exports (Art.9.6.14). This requirement can weaken or delay implementation of a new regulation, or lead to ‘regulatory chill’ where it becomes sufficiently cumbersome that it creates a disincentive to attempt a new regulation. This will have the same effects on regulatory innovation as requirements in the CPTPP, i.e. precluding or limiting novel regulations where consensus scientific evidence does not yet exist. Indeed, both the USMCA’s and CPTPP’s emphases on ‘science-based risk management’ undermine the ‘precautionary principle’ (when evidence is unclear or the policy process prone to regulatory capture, err on the side of caution), a principle which remains embedded in EU law (and its trade policy) and is considered an important public health ethic [37].

Curiously, the USMCA, as does the CPTPP, acknowledges “qualitative and quantitative information” as “scientific data” (Art.9,6.9). This calls into question the old NAFTA Chapter 11 ISDS *Bilcon* case, in which 2 of 3 tribunalists in their ruling against Canada and in favour of the investor stated that “community concerns (core values)”, very much a qualitative assessment, should not have been considered in the assessment review.^5^ Finally, the USMCA in a new article (not found in other SPS texts) calls on Parties “to endeavor to enhance the compatibility of its sanitary and phytosanitary measures with [those] of the other Parties” up to and including identical measures (Art.9.7.2). This is a soft requirement (“endeavor to enhance”) and there is some flexibility in the proviso that SPS compatibility should “not reduce each Party’s appropriate level of protection.” But it is indicative of the move towards continent-wide similar/identical standards, which begs the question: which of the three Parties holds the most economic cards in such an endeavor?

#### Good regulatory practice

Chapter 28 of the USMCA is modelled on the novel regulatory coherence chapter of the CPTPP, but the USMCA commitments are more extensive and more binding legal language is used throughout. In contrast to the CPTPP, its implementation is enforceable through state-to-state dispute settlement, at least to “address a sustained or recurring course of action or inaction that is inconsistent with a provision of this Chapter” (Art. 28.20). The CPTPP’s regulatory coherence chapter has been described as a significant step in embedding provisions in trade agreements that not only commit Parties to cooperate on regulatory matters and to enhance the compatibility of regulations, but also to specify how regulations should be developed at the domestic level [39]. The USMCA takes the regulatory coherence agenda to another level with commitments that are more detailed and prescriptive, and legal language that is more forceful (e.g. Parties ‘shall’ rather than ‘should’ abide by the chapter’s different provisions). While the CPTPP allows each Party to determine the scope of regulatory measures that would be covered by its regulatory coherence chapter, USMCA text has no such provision essentially placing all regulatory measures within its ambit.

While Art 28.2.3(a) states that the chapter does not prevent a Party from “pursuing its public policy objectives (including health, safety and environmental goals), at the level it considers to be appropriate,” several provisions in the chapter could make it difficult for Parties to do so. Art.28.9, for example, requires authorities developing regulations to publish the regulation, along with detailed explanatory information, before the regulation is finalised, and in time for comments to be received and acted upon from “any interested person, regardless of domicile.” Art.28.11 provides a long list of requirements that should be observed when undertaking regulatory impact assessments; while Art. 28.14 obliges Parties to ensure opportunities for “any interested persons” to make “written suggestions for the issuance, modification, or repeal of a regulation” if, among other reasons, it has become more burdensome than necessary to achieve its objective “(for example with respect to its impact on trade)”, providing a mechanism by which regulated industries can petition governments to deregulate.

While some of these requirements reflect the existing regulatory practices in some countries (such as the US), including them as binding commitments in a trade agreement potentially provides grounds for complaints by industry (by persuading the host state to complain) that the proper process has not been followed in developing regulations. The administrative compliance burden imposed by the USMCA (greater than that in the CPTPP) risks bogging down in ‘red tape’ development of new health, safety, or environmental protective measures. The Parties “shall”, for example, adopt or maintain processes for internal consultation, coordination and review in the development of regulations (Art. 28.4), whereas the CPTPP text only states that they should do so, and explicitly recognises that there will be differences in regulatory approaches between Parties. USMCA Parties must annually publish lists of proposed regulations (Art 28.6), provide a dedicated website for information about regulations that are being developed (Art 28.7), publish more detailed information about regulations once they are finalised (Art 28.12) and about the processes used by regulatory authorities “to prepare, evaluate or review regulations” (Art 28.15), and “adopt or maintain procedures or mechanisms to conduct retrospective reviews of its regulations” (Art 28.13).

Article 28.10 of the USMCA requires each Party to “encourage its regulatory authorities to ensure that the membership of any expert group or body” that is providing scientific or technical advice to a regulatory authority “includes a range and diversity of views and interests, as appropriate to the particular context”. While the Framework Convention on Tobacco Control (FCTC) makes clear that it is not appropriate for Parties to involve the tobacco industry in policy development -- effecting shielding tobacco control policy from this USMCA obligation -- whether the expert groups and bodies in other public health policy areas such as food, alcohol and pharmaceutical policy can be similarly quarantined from industry membership in the absence of an FCTC-like instrument remains to be seen.

#### Investor-state dispute settlement

One of the more contentious aspects of recent trade agreements is inclusion of chapters that extend special rights and protections to foreign investors. While bilateral investment treaties initially were intended to encourage foreign investment in low- and middle-income countries whose domestic courts were feared liable to political capture if governments decided to nationalize investors’ assets, investor-state dispute settlement (ISDS) provisions quickly became standard in FTAs. NAFTA was the first regional trade agreement to incorporate more expansive investor rights beyond direct expropriation to include ‘indirect expropriation’, where government measures could affect the value (including anticipated future profitability) of an investment. The number of investor claims and amount of awards determined by tribunals also increased dramatically since the 1990s, often with the encouragement of corporate law firms profiting from such challenges [40]. Many of these challenges relate to health, environment, and social protection measures, and almost half of recent disputes have been between high-income countries with few concerns over the transparency or accountability of their court systems [40]. Under NAFTA, for example, US investors sued Canada 26 times and Mexico 17 times, while Canadian investors sued the US 15 times, and Mexican investors only once [41]. While the US never lost a NAFTA ISDS case, 4 of the suits against Canada ruled in favour of investors, 5 were settled before arbitration, and 5 are still outstanding as of May 2019 [42]. Procedurally, ISDS tribunals have been criticized for a lack of transparency, conflicts of interest amongst arbitrators ruling on disputes, and lack of appeal processes. Criticism of ISDS have led to some amendments in recent agreements. The CPTPP, for example, limits (although does not entirely eliminate) claims for ‘indirect expropriation’, allows governments “to elect to deny the benefits” of ISDS for any claims against tobacco control measures or on contracts foreign investors enter into with governments, and some CPTPP Parties have signed ‘side letters’ further restricting ISDS use [43].

Given the role played by NAFTA in promoting expansive ISDS provisions in subsequent treaties, it is significant that the USMCA has significantly limited the application of ISDS provisions in its investment chapter. Annex 14-D still allows ISDS between the US and Mexico, albeit with far more limited grounds for making a claim (indirect expropriation, for example, is ruled out as the basis for claim). Under Annex 14-E, however, US-initiated ISDS claims related to five of Mexico’s specific sectors (oil and natural gas, power generation, telecommunications, transportation services, and some infrastructure) will be able to rely on the full set of investor protections included in Chapter 14 (including indirect expropriation). This has environmental and human health implications to the extent it precludes or “chills” future Mexican regulations to limit fracking or oil drilling. Annex 14-C also allows for “legacy investment claims”, meaning any claim made by an investor within 3 years of the “termination of NAFTA 1994”, which will occur if the USMCA enters into force, likely sometime in 2019. Legacy challenges filed within this period will be allowed to continue under old NAFTA provisions until a final decision is made by an arbitration panel. New ISDS claims were already filed even before the USMCA was signed to take advantage of this provision, with some analysts fearing a rush of new claims before the 3 year period expires. But as more than one commentator has noted:

The dramatic turnaround on ISDS in the renegotiated NAFTA, the agreement which triggered the explosion of ISDS cases in the late 1990s, is significant. Perhaps it will once again spur a novel approach to international investment governance, but one in which ISDS is no longer the norm or is at least more tightly circumscribed [44].

### Labour and environmental Protection

Labour provisions within FTAs can impact health in a number of ways [45]. For example, provisions that shift distributions of power among economic actors (e.g. organized labour, governments and business/corporate actors) can shape employment conditions such as labour standards, occupational health, safety regulations and union protections. Provisions can also deal directly with employment conditions which impact health through exposure to risk factors, injuries, physical and chemical hazards, ergonomics, psychosocial conditions and broader determinants of health related to wages, economic security and social inequalities.

The inclusion of labour and environmental chapters in regional trade agreements (there are none in the WTO) is often lauded by governments negotiating such agreements that workers’ rights and environmental protection will be safeguarded even as commercial trade between countries increases. Given the importance of working conditions and the sustainability of our ecological commons are to human health, such attention in trade agreements should be welcomed by public health. Although such provisions may slow down a regulatory race to the bottom, however, they often do little more than simply entrench an existing and not terribly health-equitable status quo.

#### Labour

Like other areas of the USMCA, many of the provisions in its Labor Chapter are modelled on the CPTPP, which we have reviewed in detail elsewhere [45]. As with other labour chapters (including in the CPTPP), the USMCA refers only to the ILO Declaration and not to any of its more specific, fundamental Conventions (of which the US has only ratified 2, and neither of those related to workers’ right to organize and collectively bargain); and which, if implemented and enforced, contribute both directly and indirectly to workers’ health. Also like the CPTPP, and despite several incremental improvements, much of the language surrounding labour standards in the USMCA is hortatory. The acceptability of labour standards, for example, is to be determined by each individual country.

The USMCA also neglects important labour rights, notably those pertaining to social protection policies which can provide a means of mediating the health impact of job loss, for example, through unemployment insurance [46]. In Mexico, there is currently no unemployment benefit anchored in national legislation. While Canada and the US both have legislated unemployment schemes, the share of unemployed who actually receive benefits is 40 and 27.9%, respectively [47]. Through its Trade Adjustment Assistance program, the US is unique in offering targeted social protection for trade displaced workers, including job retraining services. The scheme, however, requires workers to submit a joint petition to the Department of Labor—leading to long delays in benefit receipt. Historically, it has also covered only a minority of workers displaced by trade [48] and has failed to secure new employment for the vast majority of those completing its retraining services [49].

More positively, the chapter’s article on forced labour was strengthened from the CPTPP text so that parties “shall prohibit” (rather than “shall. .. discourage”) importation of goods produced by forced labour. There are also two new articles in the chapter which cover violence against workers generally (23.7) and migrant workers, specifically (23.8), along with a strongly worded provision on gender equity not found in the CPTPP:

Accordingly each Party shall implement policies that protect workers against employment discrimination on the basis of sex, including with regard to pregnancy, sexual harassment, sexual orientation, gender identity, and caregiving responsibilities, provide job-protected leave for birth or adoption of a child and care of family members, and protect against wage discrimination. (Art.23.9.1).

While the Trump administration’s latest move against gender identity or sex identity already violates this provision, a footnote to this provision specifically says the US’s current policies regarding hiring is “sufficient to fulfill the obligation set forth.” This addition is likely the result of the 40 Republican members of Congress who, in a letter to President Trump, said a trade deal was “no place for the adoption of social policy” [50]. The labour chapter also requires that Mexico allow independent trade unions, weakening the present domination of non-independent unions that work more in corporate than worker favour. This is a positive step forward, and one the new government in Mexico is likely to follow, although there are indications that Mexico’s 2019 budget may be unlikely to include funding to implement these labour reforms [51].

As with each government’s discretion in setting its own labour standards, each country retains sovereignty over setting its enforcement procedures. There is a certain irony, for example, that the US under the Obama administration amended its policy that had allowed importing goods made by prison labour if domestic supply did not meet ‘consumptive demand’, and continues to include clauses banning imports of goods made by prison labour, while allowing its own companies to employ prison labour, as the following describes:

Prisoners earn as little as 23 cents an hour working on assembly lines, and their products often compete for business with legitimate government contractors who operate under the Berry Amendment (a law that requires U.S. military uniforms to be made in the U.S.). These respectable American companies make apparel and footwear for many branches of the U.S. government and often use the Berry business as an anchor, to help them run their U.S. factories so they can compete in the global marketplace [52].

The extent to which such clothing is directly or indirectly exported (or passed through firms in a way that disguises its origin in prison labour) is unknown. Even if such apparel remains for domestic consumption only, there is an arguable case that the US ban on imports made by prison labour ironically violates national treatment provisions.

As with the CPTPP, violations of labour conditions with the USMCA are confined only to circumstances where a labour right is denied or level of labour protection lowered in order to obtain a trade or investment advantage. This would seem to exclude all persons working in the non-export or private (investment-driven) sectors, such as public employees and teachers, with potential knock-on effects for health equity [45]. However, for the first time in a trade agreement, the USCMA chapter does provide a definitions of the oft-used phrases “through a sustained or recurring course of action or inaction” and “in a manner affecting trade between the Parties”:

For greater certainty, a “sustained or recurring course of action or inaction” is “sustained” where the course of action or inaction is consistent or ongoing, and is “recurring” where the course of action or inaction occurs periodically or repeatedly and when the occurrences are related or the same in nature. A course of action or inaction does not include an isolated instance or case (footnote 8).

For greater certainty, a “course of action or inaction” is “in a manner affecting trade or investment between the Parties” where the course involves: [1] a person or industry that produces goods or provides services traded between the Parties or has investment in the territory of the Party that has failed to comply with this obligation; or [2] a person or industry that produces goods or provides services that compete in the territory of a Party with goods or services of another Party (footnote 9).

It has been argued that these definitions set a low threshold for claims of violations and could make it easier to initiate labour-related arbitrations in the future [53]. This is important since this phrasing is precisely the point on which the only dispute to date on labour chapter provisions failed, (under the Dominican Republic-Central America Free Trade Agreement) where violations of Guatemalan labour laws were not proven to have led to a trade or investment advantage.

It is also worth noting that while this text sets limits to the obligations surrounding the protection of labour rights, there are no such limits in several other areas of the USMCA, for example, in relation to intellectual property rights where arbitration can be sought regardless of whether alleged violations affected trade. Such a course of logic, as in other areas of the Chapter, affords a higher priority to trade than to labour rights.

A further watering down of the chapter can be found in Article 23.5.3:

If a Party fails to comply with an obligation under this Chapter, a decision made by that.

Party on the provision of enforcement resources shall not excuse that failure. Each Party retains the right to exercise reasonable enforcement discretion and to make bona fide decisions with regard to the allocation of enforcement resources between labor enforcement activities among the fundamental labor rights and acceptable conditions of work enumerated in Article 23.3.1 and Article 23.3.2, provided that the exercise of that discretion, and those decisions, are not inconsistent with its obligations under this Chapter.

The value of this chapter must also be seen in light of text within the Appendix to Annex 4-B that specifies, in order to avoid US import tariffs, 40–45% automotive content (including parts and assembly) must be in jobs paying US$16/h, most of them in manufacturing (factory) employment. This arrangement represents a first attempt at integrating a guaranteed minimum wage into a trade agreement. Some trade analysts argue that a lower disparity between US and Mexican automotive workers may redirect investment (and employment) away from Mexico to the US [42]. However, it is unclear whether automakers in Mexico are likely to triple the going hourly wage, or simply opt to pay the (comparatively low) tariff (only 2.5% on cars, Mexico’s main automotive export) [54], or how adherence to this provision would be monitored by either the American or Mexican government. The provision also makes no allowance for inflation adjustment and neglects the many other economic sectors that also suffer from low wages and wage disparities.^6^

#### Environment

The Environment Chapter begins with this normative claim:

The Parties recognize that a healthy environment is an integral element of sustainable development and recognize the contribution that trade makes to sustainable development (Art.24.2.2).

No evidence is offered to support this claim, despite a joint WTO/UNEP report in 2009 acknowledging the link between increased trade and climate change [56, 57], and extensive criticisms from environmental groups on successful trade challenges to environmental protection measures [58]. More recently both organizations have begun a dialogue to document how international trade might be conducted such that it can also ensure “a healthier environment” [59].

The USMCA recognizes that each Party has “the sovereign right” to establish its “own levels of domestic environmental protection” and the right to modify this as it sees fit, while calling on Parties to “strive to ensure” (weak language) that its laws provide for “high levels of environmental protection.” This is language similar to that in the CPTPP and other FTAs, along with the proviso that “No Party shall fail to effectively enforce its environmental laws *through a sustained or recurring course of action or inaction in a manner affecting trade or investment between the Parties*…” (Art.24.4). This is the same language that weakens provisions in the labour chapter and renders much of the rules in the environment chapter first, because it is difficult to prove “sustained and recurring” and, second, because the concern of this chapter is not with environmental protection, but (as with the labour chapter) lowering standards specifically to gain a trade or investment advantage. If a country gains no such advantage, it can destroy the environmental commons at will.

Art.24.7.1 calls on Parties to undertake environmental impact assessments “of proposed projects…that may cause significant effects,” which appears to be a novel addition to a trade agreement but, since all three countries already have domestic requirements for such impact assessments, what it means and why it is here in a trade treaty is unclear. The chapter repeats calls in other treaties for cooperation between the Parties with respect to engaging in new multilateral environment agreements, notably:

The Parties commit to consult and cooperate as appropriate with respect to environmental issues of mutual interest, in particular trade-related issues, pertaining to relevant multilateral environmental agreements. This includes, inter alia, exchanging information on the implementation of multilateral environmental agreements to which a.

Party is party; *ongoing negotiations of new multilateral environmental agreements; and, each Party’s respective views on becoming a party to additional multilateral environmental agreements* (Art.24.8.3)*.*

The italicized text may be insignificant, but it could also lead to pressures from one Party on another if its implementation or new MEA negotiations do not align with the trade/investment or economic interests of the other Party. The language here is stronger than in the CPTPP, which only identifies a need for dialogue between the Parties; the USMCA *commits* Parties to such cooperation and information sharing.

There are some mixed elements, largely in how the chapter references several MEAs, such as the *Montreal Protocol* on ozone-depleting substances (Art.23.9), and pollution due to marine shipment (Art. 23.10). Failure of a Party to abide by these environmental accords are subject to dispute but, once more, only if existing standards are lowered to gain a trade or investment advantage. Two other environmental concerns (air pollution and ‘marine litter’, meaning micro plastic waste) are identified as serious problems but contain no specific measures, rendering them de facto unenforceable. Reference is also made to the importance of protecting biodiversity but there is no mention of the International Convention on Biodiversity, which the US (despite past programs protecting biodiversity) has yet to ratify. The Chapter also emphasizes voluntary environmental measures (Art.24.14), but with the usual *caveat* that even here such measures should “avoid the creation of unnecessary barriers to trade.”

Other articles, however, do reference several other environmental issues:

- protection of ‘wild fisheries’ (24.17)
- ‘fisheries management’ (24.18 – though the language here is softer, ‘shall seek to’ rather than ‘shall’)
- ‘conservation of marine species’ (24.19, which bans killing of great whales) and,
- as in the CPTPP, forceful language banning subsidies for fishing fleets that work in overfished waters (24.20)

Art. 24.23, like other references to environmental issues, recognizes the importance of maintaining healthy forests by emphasizing harvesting of legal logs only, which begs the question how effective are such laws and their enforcement, and fails to account that legal logs are likely monocrop industrial forests which pose severe risks to biodiversity. Finally, Art.24.22 also requires governments to implement obligations under the Convention on International Trade in Endangered Species of Wild Fauna and Flora (CITES). The Convention does not ban trophy hunting (poaching, illegal trade, and habitat loss/climate change are far greater threats to wildlife) but does monitor annual import/export permits for such trophy animals to determine if such hunting is posing danger to species’ populations. The problem with Article 24.22, however, is that (as in many of the provisions in this chapter) it is subject to dispute only if the lowering of government standards creates a trade or investment advantage.^7^

### Exceptions and general provisions and dispute resolution

The USMCA contains the same language around exceptions as in GATT XX (b) and GATS XIV (b), which outline a multi-staged necessity rules for exceptions for health and environmental protection. Although WTO dispute panels have acknowledged the importance of the health exception in recent decisions, national treatment (non-discrimination) and ‘least trade restrictive’ requirements in trade rules have made it difficult for countries to use these exceptions to defend health regulatory measures. The most contentious issue under the USMCA, however, is Article 32.10, which requires any Party entering negotiation with a ‘non-market country’ (aka China) to notify and consult with other Parties in advance. This is widely seen as the US ensuring that Canada and Mexico follow the US lead in trade or investment dealing with China. Failure to abide by this could lead to termination of the USMCA by one of the two other Parties (Article 32.10.4).

All regional trade agreements contain rules governing the settling of disputes between Parties. Chapter 31 of the USMCA follows a pattern similar to other regional agreements, i.e. that Parties can file for dispute under WTO dispute settlement rules, or under those established in the USMCA Chapter. The USMCA does contain some positive developments, including making panel hearings public unless confidential material is being discussed. Although there is a cumbersome process of consultation before a dispute panel might be established (up to 3 months or longer), panelists are drawn from a roster (similar to the yet-to-be ratified Investor Court System of the Canada/EU Comprehensive Economic and Trade Agreement, or CETA) and are subject to a code of conduct (similar to that proposed for the dispute settlement panels of the CPTPP, but without, as yet, any definition of the content of such a code). The improvements made by the USMCA are that a dispute panel concerning a labour issue must include a panelist with expertise in labour law; with the same applying to a dispute on an environmental issue (i.e. a panelist with expertise in environmental law) (Art.31.8.3). If two Parties are involved in the same dispute, the panel is increased from three to five members (presumably allowing both disputing parties to elect from the roster their panelist). Art.31.11 further requires panelists to consider requests from NGOs in the countries involved in the dispute (*amicus curiae* briefs) but, as with other such trade treaties, there is no binding requirements that panelists incorporate such briefs in their findings. A weakness in the USMCA, however, is that unlike the WTO system, there is no Appellate Body, although a panel may consider a rebuttal (appeal) from a disputing Party. This is not surprising given the US Trump Administration’s persistent blocking of nominees to the WTO Appellate Body, risking its imminent collapse and, with it, the end of the WTO’s dispute settlement process [60].^8^

## Conclusion

Despite each Party being able to name the agreement placing their own initial first, the USMCA is the apt acronym given that the agreement is very much based on what the US wanted and was able to strong-arm from its two North American partners. The USMCA essentially uses the original TPP as its template and ratchets up the obligations as well as reinstating provisions that were suspended in the CPTPP after it had withdrawn. In many areas, flexibilities that were carefully negotiated in the TPP context (with several developing countries at the table and 10–11 countries negotiating together against the US position) have been lost in the USMCA, which will now become the template for the next US FTA, with worrying implications for the next low- or middle-income country to enter negotiations with the US. This is not a new strategy of the Trump administration; as far back as 2009 under the Obama administration, the US WTO Ambassador emphasized the importance of “sustained direct bilateral engagement” as the way to move the global trade agenda forward [61], a strategy also adopted by the EU given the inability of these wealthier nations to hold negotiating sway over the multilateral WTO system. What is new with the Trump administration is its apparent willingness to forego negotiating etiquette and, instead, to use tariffs and the threat of trade wars to dominate bilateral or regional negotiations with economically smaller or weaker countries. In its trade and hegemonic power competition with China, the US strategy may extend to undermining or withdrawing from the multilateral WTO in pursuit of bilateral agreements where it can more easily achieve its own trade interests.

The decision by the three Parties to limit ISDS provisions is widely regarded as a positive step in respect to protecting new health, social, or environmental protection measures from investor challenges, a somewhat paradoxical stance for the US, since US investors under NAFTA had benefitted most from ISDS rules. These rules, of course, persist as ‘legacy claims’ for some time to come, and some Mexican sectors are still very much bound by old NAFTA ISDS provisions; but it does indicate a shift in US trade and investment policy. At the same time, the new, extended, and enforceable obligations the USMCA places on regulators during policy development and implementation, effectively giving voice to corporate (investor) interests during the processes leading up to new regulations, reduces the necessity of using ISDS as a tool to threaten or challenge regulations not in their interests. Enforcing regulatory cooperation between Parties where the economic power of one (the US) outstrips the other two by a full order of magnitude will bias heavily in the regulatory direction of the one. If the US were a paragon of health, social, and environmental regulatory protection this could coerce the other two Parties to improve their own levels of such protection. This is not the case with the actual obligations under the labour and environmental chapters of the USMCA, however, and the present Trump administration appears to have more interest in weakening its past regulatory protections than in strengthening them. Indeed, USMCA provisions in its TBT, SPS and Regulatory Coherence chapters appear to follow the Trump administration’s deregulatory playbook, by significantly increasing the influence of the corporate sector in regulation-making while imposing new administrative burdens that are likely to slow or even “chill” development of new regulations.

Finally, the USMCA entrenches and extends intellectual property rights for pharmaceuticals at a time when high-cost biologics not only remain out of reach for large parts of the world’s population, but are increasingly straining pharmaceutical budgets even in wealthy countries. The USMCA reinstates TPP provisions (suspended in the CPTPP) that encroach on pharmaceutical policy via a range of avenues, potentially shrinking the space available to regulators to contain costs and obtain value for money, ensure drug safety and efficacy, and shield pharmaceutical policy from industry influence.

Bottom-line: the USMCA is not a health-enhancing template for future FTAs that governments should emulate.

## Footnotes

1 TRIPS-Plus refers to intellectual property rights which extend beyond those provided for by the WTO’s Agreement on Trade-Related Aspects of Intellectual Property Rights (TRIPS).

2 Like data protection, ‘market protection’ applies to the clinical trial data submitted to regulators and commences from the date of marketing approval for the originator product. However, market protection differs from data protection in that the regulator can accept and evaluate an application for a follow-on product during the period of market protection (although marketing approval can’t be granted until the market protection period expires).

3 It is not possible to make identical generic copies of biologics due to their complexity, but biosimilars, which are highly similar to the original biologic and have the same therapeutic effect, can often be made at a lower cost than the original biologic.

4 The USMCA chapter on Good Regulatory Practice further requires Parties, in an annual retrospective review of their regulations and at the instigation of any “interested person” from any other Party, to “consider…common approaches to the display of product or consumer information”, another potential way for the US to initiate a challenge to Canadian or Mexican front-of-pack interpretative nutritional labeling.

5 A US corporation sued a provincial government after an environmental impact assessment rules against its massive quarry and marine shipping terminal, in part because it was contrary to community values. Despite the investor winning the suit, the panel, noting the quarry was unlikely ever to go ahead, denied the US$477 million investor claim for 50 years of lost profit and awarded only US$17 million for lost opportunity costs [38].

6 As with other new FTAs, such as the CPTPP, the USMCA has been promoted for the positive impact it will have on economic growth and employment creation. But there is also the question of who benefits from such an agreement. To date there have been two econometric studies attempting to estimate economic and employment gains arising from the USMCA, both focusing on the US. In April 2019 the US International Trade Commission (USITC) released a lengthy study in which it forecast gains of US$68.2 billion and 176,000 new jobs [42]. However, these simulated gains rest on several assumptions, including only slight restrictions on the ability of dislocated workers to move to other employment, and an attempt to model “reduced policy uncertainty”, a novel approach not embraced by many other economists. Remove the “reduced policy uncertainty” assumption and the US economy declines slightly, losing US$22.6 billion in value and almost 60,000 jobs. The second study, published one month earlier by the IMF and using similar econometric modelling to the USITC report but without the “reduced policy uncertainty” assumption, concluded that, at most and across all three countries, the USMCA would generate an additional US$540 million in aggregate GDP [55]. Canada and Mexico both gain while the US loses, but the amounts are so low that “the effects on members’ real GDP is negligible” (p.12). However, if the US steel and aluminum tariffs and Canadian and Mexican retaliatory taxes are all removed, the USMCA could generate an additional $US2.5 billion in aggregate welfare, most of it going to the US.

7 Other measures of the USMCA can also have negative environmental and subsequent health impacts. As with continuing ISDS provisions between the US and Mexico with respect to fracking and oil drilling, a side letter agreement between Canada and the US guarantees either country access to their pipelines for purposes of importation. This could increase oil and natural gas transmissions with subsequent risk of fugitive leaks and emissions, posing both environmental and human health risks.

8 The WTO Appellate Body (AB) is comprised of seven ‘judges’ appointed for a fixed term, and has the power to uphold, modify, or reverse a dispute panel decision. At least three members of the AB must render a final decision for any action on a dispute to be binding. The US considers the AB to be exercising ‘judicial activism’ particularly regarding disputes over anti-dumping measures, which the US has raised with regard to both China and the EU, and generally loses on appeal to the Appellate Body. The US has a particular concern over how China’s system of ‘state capitalism’ threatens its model of liberalized international trade, which it believes is the foundation of the WTO. With the US blocking new appointments to the AB, however, the WTO dispute settlement process could break down and with it, the entire multilateral trading system creating what some trade analysts characterize as a ‘return to the wild west of trade’ [60].

## References

[1] USTR’s Office of the Western Hemisphere. Western Hemisphere [Internet]. Office of the United States Trade Representative. [cited 2019 Feb 21]. Available from: https://ustr.gov/countries-regions/americas/

[2] Government of Canada. Canada-Mexico relations: a strategic partner for Canada [internet]. Canada’s international gateway. 2018 [cited 2019 Feb 21]. Available from: https://www.canadainternational.gc.ca/mexico-mexique/canmex.aspx?lang=eng.

[3] Labonté R, Schram A, Ruckert A (2016). The trans-Pacific partnership: is it everything we feared for health?. Int J Health Policy Manag.

[4] Labonté R, Schram A, Ruckert A (2016). The trans-Pacific partnership agreement and health: few gains, some losses, many risks. Glob Health.

[5] Ruckert A, Schram A, Labonté R, Friel S, Gleeson D, Thow AM (2016). Policy coherence, health and the sustainable development goals: a health impact assessment of the trans-Pacific partnership. Crit Public Health.

[6] Hirono K, Haigh F, Gleeson D, Harris P, Thow AM, Friel S (2016). Is health impact assessment useful in the context of trade negotiations? A case study of the trans Pacific partnership agreement. BMJ Open.

[7] Patented Medicine Prices Review Board. Potential Savings from Biosimilars in Canada [Internet]. Patented Medicine Prices Review Board. 2018 [cited 2019 Feb 21]. Available from: http://www.pmprb-cepmb.gc.ca/view.asp?ccid=1304.

[8] Killic B. NAFTA 2.0: U.S.-Mexico-Canada Agreement Chapter 20: Pharmaceutical Related Patent Provisions [Internet]. Washington, D.C.: Public Citizen; 2019 p. 24. Available from: https://www.citizen.org/sites/default/files/nafta-2.0-pharmaceutical-related-patent-provisions.pdf

[9] Lexchin J, Gleeson D (2016). The trans Pacific partnership agreement and pharmaceutical regulation in Canada and Australia. Int J Health Serv Plan Adm Eval.

[10] Lopert R, Gleeson D (2013). The high price of “free” trade: U.S. trade agreements and access to medicines. J Law Med Ethics J Am Soc Law Med Ethics.

[11] t’ Hoen E. Private Patents and Public Health: Changing intellectual property rules for access to medicines [Internet]. Amsterdam, The Netherlands: Health Action International; 2016 [cited 2019 Feb 21]. 194 p. Available from: http://haiweb.org/wp-content/uploads/2016/07/Private-Patents-Public-Health.pdf

[12] Sell SK. Private Power, Public Law: The Globalization of Intellectual Property Rights [Internet]. Cambridge, United Kingdom: Cambridge University Press; 2003. p. 244.

[13] Beall R, Kuhn R (2012). Trends in compulsory licensing of pharmaceuticals since the Doha declaration: a database analysis. PLoS Med.

[14] t’ Hoen EF, Veraldi J, Toebes B, Hogerzeil HV (2018). Medicine procurement and the use of flexibilities in the agreement on trade-related aspects of intellectual property rights, 2001–2016. Bull World Health Organ.

[15] Aitken M. Delivering on the potential of biosimilar medicines: the role of functioning competitive markets [internet]. New Jersey, USA: IMS Institute for Healthcare Informatics; 2016 Available from: https://www.medicinesforeurope.com/wp-content/uploads/2016/03/IMS-Institute-Biosimilar-Report-March-2016-FINAL.pdf

[16] Bagnoli P, Bergeron É. The impact of the Canada – United States – Mexico Agreement on prescription drug expenditures in Canada [Internet]. Office of the Parliamentary Budget Officer. 2019 [cited 2019 Apr 25]. Available from: https://www.pbo-dpb.gc.ca/en/blog/news/CUSMA_prescription_drugs.

[17] Rep. Schakowsky JD. Improving Access To Affordable Prescription Drugs Act [Internet]. Sect. Section 303 (a)(3), H.R.1776 Introduced, 2017. Available from: https://www.congress.gov/bill/115th-congress/house-bill/1776/text).

[18] Koivusalo M, Schrecker T, Labonté R. Globalization and policy space for health and social determinants of health. In: Globalization and health: pathways, evidence and policy. United Kingdom: Routledge; 2009. p. 105–30.

[19] Office of the United States Trade Representative. Agriculture: market access and dairy outcomes of the USMC agreement [Internet]. Executive Office of the President (Press Office). 2018. Available from: https://ustr.gov/about-us/policy-offices/press-office/fact-sheets/2018/october/united-states%E2%80%93mexico%E2%80%93canada-trade-fact

[20] International Trade Administration. Mexico country commercial guide. Leading sectors for US exports and investments: best prospect overview [internet]. Export.gov. 2018. Available from: https://www.export.gov/article?id=Mexico-Agriculture.

[21] Wise T. The impacts of U.S. agricultural policies on Mexican producers. In: Subsidizing Inequality: Mexican Corn Policy Since NAFTA [Internet]. 1st ed. Washington, D.C.: Woodrow Wilson International Center for Scholars; 2010 [cited 2019 Apr 25]. p. 163–73. Available from: https://www.wilsoncenter.org/sites/default/files/Subsidizing%20Inequality_0.pdf

[22] Scott J. Agricultural subsidies in Mexico: who gets what? In: subsidizing inequality: Mexican corn policy since NAFTA [Internet]. 1st ed. Washington, D.C.: Woodrow Wilson International Center for Scholars; 2010. p. 67–119. Available from: https://www.wilsoncenter.org/sites/default/files/Subsidizing%20Inequality_0.pdf

[23] Public Citizen’s Global Trade Watch. NAFTA’s legacy for Mexico: economic displacement, lower wages for Most, increased migration [internet]. Public Citizen; 2018 [cited 2019 Feb 21]. Available from: https://www.citizen.org/sites/default/files/nafta_factsheet_mexico_legacy_march_2018_final.pdf.

[24] Murphy S, Hansen-Kuhn K. Counting the costs of agricultural dumping [internet]. Washington, D.C.: Institute for Agriculture and Trade Policy; 2017 Jun p. 1–14. Available from: https://www.iatp.org/sites/default/files/2017-06/2017_06_26_DumpingPaper.pdf

[25] IATP. Fair prices and a bold new deal in Mexico [Internet]. Institute for Agriculture & Trade Policy. 2019 [cited 2019 Apr 25]. Available from: https://www.iatp.org/blog/201902/fair-prices-and-bold-new-deal-mexico

[26] Press Release. Strengthening North American Trade in Agriculture: United States–Mexico–Canada Trade Fact Sheet [Internet]. Office of the United States Trade Representative, Executive Office of the President; 2018. Available from: https://ustr.gov/about-us/policy-offices/press-office/fact-sheets/2018/october/united-states%E2%80%93mexico%E2%80%93canada-trade-fa-2

[27] Duffey KJ, Popkin BM (2008). High-fructose corn syrup: is this what’s for dinner?. Am J Clin Nutr.

[28] Stanhope KL, Medici V, Bremer AA, Lee V, Lam HD, Nunez MV (2015). A dose-response study of consuming high-fructose corn syrup-sweetened beverages on lipid/lipoprotein risk factors for cardiovascular disease in young adults. Am J Clin Nutr.

[29] Goran MI, Ulijaszek SJ, Ventura EE (2013). High fructose corn syrup and diabetes prevalence: a global perspective. Glob Public Health.

[30] Barlow P, McKee M, Basu S, Stuckler D (2017). Impact of the north American free trade agreement on high-fructose corn syrup supply in Canada: a natural experiment using synthetic control methods. Can Med Assoc J.

[31] Committee on Technical Barriers to Trade. Decisions and Recommendations Adopted by the WTO Committee on Technical Barriers to Trade Since 1 January 1995 (G/TBT/1/Rev.12) [Internet]. World Trade Organization; 2015. Available from: https://docs.wto.org/dol2fe/Pages/SS/directdoc.aspx?filename=q:/G/TBT/1R12.pdf

[32] North American Meat Institute (NAMI), Canadian Meat Council (CMC). NAMI CMC US-Canada RCC RFI [Internet]. Regulations.gov; 2018. Report no.: FR 2018-21765. Available from: https://www.regulations.gov/document?D=OMB-2018-0006-0007

[33] Thow AM, Jones A, Hawkes C, Ali I, Labonté R (2018). Nutrition labelling is a trade policy issue: lessons from an analysis of specific trade concerns at the World Trade Organization. Health Promot Int.

[34] Labonté R. How NAFTA will make us fat if the U.S. has its way [internet]. The conversation. 2018 [cited 2019 Feb 21]. Available from: http://theconversation.com/how-nafta-will-make-us-fat-if-the-u-s-has-its-way-94198

[35] Braun JM, Sathyanarayana S, Hauser R (2013). Phthalate exposure and children’s health. Curr Opin Pediatr.

[36] Liu S, Hammond SK, Rojas-Cheatham A (2013). Concentrations and potential health risks of metals in lip products. Environ Health Perspect.

[37] Martuzzi M (2007). The precautionary principle: in action for public health. Occup Environ Med.

[38] Withers P. Canada ordered to pay US concrete company $7M in NAFTA case [internet]. 2019 [cited 2019 Apr 25]. Available from: http://www.bilaterals.org/?canada-ordered-to-pay-us-concrete.

[39] Lin C-F, Liu H-W (2018). Regulatory rationalisation clauses in FTAs: a complete survey of the US, EU and China. Melb J Int Law.

[40] Van Harten G. Foreign investor protections in the trans-Pacific partnership [internet]. Canadian Centre for Policy Alternatives; 2016 Jun. Available from: https://www.policyalternatives.ca/publications/reports/foreign-investor-protections-trans-pacific-partnership.

[41] Sinclair S. Major Complications: the TPP and Canadian health care [internet]. Canadian Centre for policy alternatives; 2016 [cited 2017 Dec 19]. Available from: https://www.policyalternatives.ca/sites/default/files/.../2016/.../Major_Complications.p...

[42] Shikher S, Torsekar M, Semanik M, Herman P. U.S.-Mexico-Canada trade agreement: likely impact on the U.S. economy and on specific industry sectors [internet]. Washington, D.C.: United States International Trade Commission; 2019 Apr p. 379. Available from: https://www.usitc.gov/publications/332/pub4889.pdf.

[43] Buchanan K. New Zealand: agreements with five countries exclude compulsory investor-state dispute settlement processes [internet]. Bilaterals.org. 2018 [cited 2019 Feb 21]. Available from: https://www.bilaterals.org/?new-zealand-agreements-with-five

[44] IISD. USMCA Curbs How Much Investors Can Sue Countries—Sort of [Internet]. International Institute for Sustainable Development. [cited 2019 Feb 21]. Available from: https://www.iisd.org/library/usmca-investors

[45] McNamara C, Labonté R (2016). Trade, labour markets and health: a prospective policy analysis of the trans-Pacific partnership. Int J Health Serv.

[46] McNamara C (2015). Trade liberalization, social policies and health: an empirical case study. Glob Health.

[47] ILO Social Protection. World Social Protection report data 2017–2019: universal Social Protection to achieve the sustainable development goals [internet]. Social Protection.org. 201AD [cited 2019 Feb 21]. Available from: https://www.social-protection.org/gimi/RessourcePDF.action?ressource.ressourceId=54887

[48] Vijaya RM. Broken Buffer: How Trade Adjustment Assistance Fails American Workers [Internet]. New York, NY: Dēmos; 2010 [cited 2019 Feb 21] p. 1–11. Available from: https://www.demos.org/sites/default/files/publications/Broken_Buffer_FINAL.pdf

[49] D’Amico R, Schochet PZ. The Evaluation of the Trade Adjustment Assistance Program: A Synthesis of Major Findings [Internet]. Oakland, CA and Princeton, NJ: Social Policy Research Associates and M50 Mathematica Policy Research; 2012. Available from: https://www.mathematica-mpr.com/our-publications-and-findings/publications/the-evaluation-of-the-trade-adjustment-assistance-program-a-synthesis-of-major-findings

[50] Select Members of Congress. Select Members of Congress to President Trump [Internet]. Congress of the United States; 2018 [cited 2019 Feb 21]. Available from: https://lamborn.house.gov/uploadedfiles/final_letter.pdf

[51] World Trade Online. Mexico’s 2019 budget proposal has no funds for labor reform. World trade online [internet]. 2019 Jan 15; Available from: https://insidetrade.com/daily-news/mexicos-2019-budget-proposal-has-no-funds-labor-reform

[52] Helfenbein R. Prisoners compete for a share of “made in USA” [Internet]. The Hill. 2016 [cited 2019 Feb 21]. Available from: https://thehill.com/blogs/pundits-blog/finance/278886-prisoners-compete-for-a-share-of-made-in-usa

[53] Claussen K. Guest Post: The U.S.-Mexico-Canada (AKA the New NAFTA) Trade Deal: Labor [Internet]. International Economic Law and Policy Blog. 2018 [cited 2019 Feb 21]. Available from: https://worldtradelaw.typepad.com/ielpblog/2018/10/guest-post-the-us-mexico-canada-aka-the-new-nafta-trade-deal-labor.html

[54] Gindin S. NAFTA renewed. Now what? Canadian Dimension [Internet] 2018;53(3). Available from: https://canadiandimension.com/articles/view/nafta-renewed.-now-what.

[55] Burfisher ME, Lambert F, Matheson TD. NAFTA to USMCA: what is gained? [internet]. International Monetary Fund; 2019 Mar [cited 2019 Apr 25]. Available from: https://www.imf.org/en/Publications/WP/Issues/2019/03/26/NAFTA-to-USMCA-What-is-Gained-46680.

[56] Tamiotti Ludivine, Teh Robert, Kulaçoglu Vesile, Olhoff Anne, Simmons Benjamin, Abaza Hussein (2009). Foreword. Trade and Climate Change.

[57] Bacchus J. The Case for a WTO Climate Waiver [Internet]. Centre for International Governance Innovation; 2017 Nov. Available from: https://www.cigionline.org/publications/case-wto-climate-waiver.

[58] IATP. WTO, UNEP Call for Improved Cooperation, Establishment of “Early Warning Mechanism” [Internet]. Institute for Agriculture & Trade Policy. 2000 [cited 2019 Feb 21]. Available from: https://www.iatp.org/news/wto-unep-call-for-improved-cooperation-establishment-of-early-warning-mechanism

[59] UNEP Press Release. UN Environment and WTO launch dialogue on healthier environments through trade [Internet]. United Nations Environment Programme. 2018 [cited 2019 Feb 21]. Available from: https://www.unenvironment.org/news-and-stories/press-release/un-environment-and-wto-launch-dialogue-healthier-environments

[60] Fabry E, Tate E. Saving the WTO Appellate Body or Returning to the Wild West of Trade? [Internet]. Institute Delors; 2018 Jun. Report No.: No. 225. Available from: http://institutdelors.eu/wp-content/uploads/2018/05/SavingtheWTOAppellateBody-FabryTate-June2018.pdf

[61] U.S. Mission in Geneva. Ambassador Kirk Gives Closing Statement at 7th Session of WTO Ministerial Conference [Internet]. U.S. Mission to International Organizations in Geneva. 2009 [cited 2019 Feb 21]. Available from: https://geneva.usmission.gov/2009/12/03/closing-statement-wto/

