# Correction to: USMCA (NAFTA 2.0): tightening the constraints on the right to regulate for public health

**DOI:** 10.1186/s12992-019-0482-x

**Published:** 2019-07-01

**Authors:** Ronald Labonté, Eric Crosbie, Deborah Gleeson, Courtney McNamara

**Affiliations:** 1 School of Epidemiology and Public Health, University of Ottawa, 600 Peter Morand Crescent, Ottawa, Ontario K1G 5Z3 Canada; 2 School of Community Health Sciences, Ozmen Institute for Global Studies, University of Nevada Reno, 1664 N. Virginia Street, Reno, NV 89557-0274 USA; 3 School of Psychology and Public Health, La Trobe University, Bundoora, VIC 3086 Australia; 4 Department of Sociology and Political Science, Norwegian University of Science and Technology, NO-7491 Trondheim, Norway

**Correction to: Globalization and Health (2019) 15:35.**

**https://doi.org/10.1186/s12992-019-0476-8**

Following publication of the original article [1], the authors flagged an error concerning two missing article references, which were unfortunately not provided prior to publication of the article.

The two missing references are:

Firstly:

Lexchin, J. (2019) Increase in drug spending in Canada due to extension of data protection for biologics: a descriptive study. Healthcare Policy 14 (3) 10–18.doi:10.12927/hcpol.2019.25796

This should be referenced under the sub-heading ‘*Pharmaceuticals and access to medicines*’ in place of the first two (under this sub-heading) references to reference ‘9’.

Secondly:

O’Brien P, Gleeson D, Room R, Wilkinson C. Marginalising health information: implications of the Trans Pacific Partnership for alcohol labelling. Melbourne University Law Review. 2017;41(1):341–91.

This should be referenced under the sub-heading ‘*Agriculture*’ in place of the second (under this sub-heading) reference to reference ‘30’.

The authors apologize for this error.
